# Protective effects of *N*-acetylcysteine on cisplatin-induced oxidative stress and DNA damage in HepG2 cells

**DOI:** 10.3892/etm.2014.2019

**Published:** 2014-10-13

**Authors:** FUGEN WANG, SHOURONG LIU, YIQIN SHEN, RANGXIAO ZHUANG, JIANJUN XI, HONGYING FANG, XUWAN PAN, JINGJING SUN, ZHAOBIN CAI

**Affiliations:** The Xixi Hospital of Hangzhou Affiliated to Zhejiang University of Traditional Chinese Medicine, Hangzhou, Zhejiang 310023, P.R. China

**Keywords:** *N*-acetylcysteine, DNA damage, oxidative stress, cisplatin

## Abstract

Hepatocyte injury is a common pathological effect of cisplatin (CDDP) in various solid tumor therapies. Thus, strategies for minimizing CDDP toxicity are of great clinical interest. *N*-acetylcysteine (NAC), a known antioxidant, is often used as an antidote for acetaminophen overdose in the clinic due to its ability to increase the levels of glutathione (GSH). In the present study, the aim was to investigate the protective effects of NAC against CDDP-induced apoptosis in human-derived HepG2 cells. The results showed that upon exposure of the cells to CDDP, oxidative stress was significantly induced. DNA damage caused by CDDP was associated with cell apoptosis. NAC pre-treatment significantly reduced the malondialdehyde (MDA) levels and ameliorated the GSH modulation induced by CDDP. NAC also protected against DNA damage and cell apoptosis. These data suggest the protective role of NAC against hepatocyte apoptosis induced by CDDP was achieved through the inhibition of DNA damage and alterations of the redox status in human derived HepG2 cells. These results indicate that NAC administration may protect against CDDP-induced damage.

## Introduction

*N*-acetylcysteine (NAC) is an excellent source of sulfhydryl groups and is rapidly absorbed following an oral dose. The well-known role of NAC is as an antidote to acetaminophen toxicity; it has been used for this purpose for >30 years (1). It is also widely used to treat chronic obstructive pulmonary disorder, pulmonary fibrosis, and contrast-induced nephropathy. NAC also appears to have beneficial effects in conditions such as liver injury, cancer, heart disease, HIV infection and cigarette smoking (2,3). *In vitro*, NAC that has been deacetylated by cytosolic esterases in cells, causes an increase in the concentration of dissociated cysteine and an increase in glutathione (GSH) synthesis at a concentration of 5 mM (4,5). *In vivo*, NAC is actively transported into hepatocytes and converted into metabolites capable of stimulating GSH synthesis. GSH, a tripeptide synthesized from glutamic acid, cysteine and glycine via GSH synthetase and L-glutamate-cysteine ligase, plays an pivotal physiological role in the maintenance of redox status. During conditions of oxidative stress, GSH is depleted in cells, and its biosynthesis is limited by the rate of cellular cysteine uptake (6,7). Effective treatment with NAC provides sufficient cysteine to promote detoxification and directly eliminate reactive oxygen species (ROS). NAC also modulates the inflammatory response through signaling pathways that control pro-inflammatory nuclear factor (NF)-κB activation (8,9).

The chemotherapeutic agent cisplatin (CDDP) is a potent drug that has successfully been used clinically to treat cancers of the lungs, testis, ovary, cervix and genitourinary tract (10,11). The biological activity of CDDP is founded on the formation of covalent products with nucleic acids that interrupt DNA replication, transcription and ultimately, cell cleavage that finally leads to cell death. However, the clinical use of CDDP in chemotherapy is hampered by its severe side-effects. There is evidence that oxidative stress is involved in the liver damage that occurs following the administration of CDDP. A number of studies have demonstrated that CDDP-induced cell damage is contributed to by several ROS (12–15). Thus, the protective effect of free radical scavengers may prevent the generation of ROS and block the downstream cascade that leads to cell apoptosis.

Our previous study *in vivo* showed that NAC raised the antioxidant capacity of hepatic tissue in rats administered alcohol (16). However, considering the conspicuous effects of NAC *in vivo*, the protective roles observed for CDDP have not been completely investigated. Therefore, the objective of the present study was to investigate whether beneficial effects are observed *in vitro* with NAC when cells are exposed to CDDP. Thus, CDDP was employed as damage inducer, and the protective effects of NAC were evaluated through a cell viability assay, apoptosis assay, single cell gel electrophoresis (SCGE) assay and the measurement of biochemical parameters related to redox status in HepG2 cells.

## Materials and methods

### Chemical products

NAC (CAS No. 616-91-1) and dimethyl sulfoxide (DMSO, CAS No. 67-68-5) were purchased from Sigma-Aldrich (St. Louis, MO, USA). CDDP (*cis*-diamminedichloroplatinum(II), CAS No. 15663-27-1) was purchased from Melone Pharmaceutical Co., Ltd. (Dalian, China). All other chemicals were analytical grade products.

### Cell culture and treatments

HepG2 cells were provided by Keygen Biotech Co., Ltd. (Nanjing, China). The cells were stored in liquid nitrogen. All experiments were performed within the third and fifth passages. Cells were grown in a complete cell cycle in Dulbecco’s modified Eagle’s medium (DMEM; Gibco Life Technologies, Carlsbad, CA, USA) with 10% heat-inactivated fetal bovine serum (FBS; Hangzhou Sijiqing Biological Engineering Materials Co., Ltd., Hangzhou, China) and 1% penicillin/streptomycin antibiotic mixture (Gibco Life Technologies) in culture flasks (25 cm^2^) in a humidified incubator (Thermo Fisher Scientific, Waltham, MA, USA) with an atmosphere of 95% air and 5% CO_2_ at 37°C. For subcultivation, cells were passaged when they reached 80–90% confluency in the flask. The cells were trypsinized, washed with phosphate-buffered saline (PBS; pH 7.4) and centrifuged (450 × g, 5 min). Cell viability was determined by staining the cells with trypan blue.

To evaluate the impact of the CDDP on cell viability, the cells were exposed to CDDP (1.0, 2.0, 4.0, 8.0 μM) for a period of 24, 48, 72 h and then were analyzed by 3-(4,5-dimethylthiazol-2-yl)-2,5-diphenyltetrazolium bromide (MTT) assay. Cells incubated with culture medium alone were used as a negative control. HepG2 cells were pre-treated for 3 h with various concentrations of NAC (50.0, 100.0, 200.0 μM) prior to exposure to CDDP (2.0 μM) for 48 h.

Cell lysates were formed with 0.1% sodium dodecyl sulfate solution in PBS for determination of the biochemical parameters. The cell suspensions were incubated for 30 min on ice and centrifuged at 550 × g for 10 min (4°C). Subsequently, the supernatants were used for the assessment of the biochemical parameters associated with redox status. In all experiments, solvent controls were included, and NAC and CDDP were dissolved in DMEM.

### Cell viability assessment

Cell viability was evaluated biochemically with the MTT assay (17). The HepG2 cells were seeded at ~1×10^5^ cells/ml into the wells of a 96-well plate in complete DMEM, then allowed to attach and recover for ≥24 h prior to assay. Different concentrations of NAC and CDDP were added to each well. Following treatment, the medium was carefully replaced with 200 μl DMEM without FBS for 1 h at 37°C. Then, 20 μl 5 mg/ml MTT was added to each well and the cells were incubated for a further 4 h. The MTT solution was removed and 150 μl DMSO was added to lyse the cells. The components of the wells were mixed thoroughly with shaking until the crystals were completely dissolved. The absorbance at 570 nm was measured using a microenzyme-linked immunosorbent assay (ELISA) reader (model 680; Bio-Rad Laboratories, Hercules, CA, USA). The reduction in viability of the cells was expressed as a percentage compared with the viability of the control group, which was designated a viability of 100%. Each experiment was repeated three times in triplicate samples.

### Cell apoptosis assay

In order to determine the effects of CDDP on HepG2 cell apoptosis, annexin V/propidium iodide (PI) staining was performed and analysis was conducted by flow cytometry (FACSCalibur; BD Biosciences, San Jose, CA, USA), following the instructions of the annexin V FITC apoptosis detection kit (BD Biosciences). Briefly, cells were harvested, washed twice with precooled PBS and resuspended in 500 μl binding buffer following treatment with different concentrations of CDDP and NAC. Then, the cells were stained with 5 μl annexin V-FITC and 5 μl PI for 15 min at room temperature in the dark. The cell suspension was analyzed by flow cytometry. A blue (488 nm) excitation laser was used for both FITC and PI emission channels. The measured results were analyzed using CellQuest version 3.3 software (BD Biosciences).

### SCGE assay

The SCGE assay was performed according to the procedure described by Singh *et al* (18). Following treatment with different concentrations of CDDP and NAC, HepG2 cells were washed twice with PBS, trypsinized for 3 min, and suspended in 1 ml PBS. Cell suspensions were used for the determination of DNA damage. Frosted glass slides were coated previously with 100 μl normal melting agarose (NMA, 1%) and refrigerated to gel the agarose at 0°C for 5 min. For each slide, 75 μl low melting agarose (LMA, 0.65%) was mixed with 5–10 μl single cell suspension at 37°C. All the slides were covered with coverslips and refrigerated to gel the agarose in an ice box. After ~10 min, the coverslips were removed. Then, the slides were coated with 75 μl LMA (0.65%) and the agarose was solidified. The slides were then placed in a closed box containing freshly prepared cell lysis solution (2.5 M NaCl, 100 mM EDTA, 10 mM Tris HCl, 0.5% sodium *N*-lauroyl sarcosinate, adjusted to pH 10.0 with NaOH, 1% Triton X-100 and 10% DMSO) at 4°C for 1 h. Alkaline unwinding was carried out for 20 min in a horizontal DYCP-38C electrophoresis chamber (Beijing Liuyi Instrument Factory, Beijing, China), which was filled with freshly precooled alkaline buffer (300 mM NaOH, 1 mM EDTA), and electrophoresis was performed in the same buffer for 20 min at 300 mA, 20 V. The slides were then neutralized three times with a neutralization buffer (0.4 M Tris, pH 7.5) for 5 min. Then, the slides were immersed in methanol for 3 min and stained with GelRed (Biotium Inc., CA, USA).

Thereafter, the slides were covered with coverslips and analyzed immediately. The following steps were carried out in the dark. The SCGE results were examined at a magnification of 200 using a BX51 fluorescence microscope (Olympus Corporation, Tokyo, Japan), which was equipped with an excitation filter at 590 nm. Images of the comets were captured by an Olympus DP50 digital camera. The CASPLab analysis system (http://mailbox.univie.ac.at/christoph.helma/comet/) was employed to measure various comet parameters. The results were expressed as tail length, tail moment and olive tail moment in 100 randomly selected comet cells.

### Measurement of GSH, malondialdehyde (MDA) and superoxide dismutase (SOD)

The GSH level was measured by the addition of 5,5′-dithio-bis(2-nitrobenzoic acid) (DTNB). DTNB, a symmetric aryl disulfide, reacts with thiols to yield a yellow colored chromophore; this product can be quantified by its maximum absorbance at 412 nm. Concentrations of GSH were determined from a freshly prepared standard curve. The measurements were conducted with a model UV-2550 spectrophotometer (Shimadzu Co., Ltd., Tokyo, Japan). The protein concentrations of the supernatant were determined using a bicinchoninic acid (BCA) protein assay kit (Nanjing Jiancheng Bioengineering Institute, Nanjing, China).

Lipid peroxidation was evaluated by measuring MDA concentrations according to the thiobarbituric acid (TBA) method. This method involved the spectrophotometric measurement of the red color produced during the condensation reaction of MDA with thiobarbituric acid. The reactive products were quantified by the absorbance at 532 nm. MDA concentrations were expressed in terms of nmol/mg protein.

SOD activity was measured using a kit (Nanjing Jiancheng Bioengineering Institute), based on the ability of SOD to inhibit the conversion of WST-1 to a formazan dye by superoxide anions produced from a xanthine-xanthine oxidase system. One unit of SOD activity was defined as the amount that caused a 50% reduction in the absorbance at 450 nm. The amount of SOD was expressed in units of U/mg protein.

### Statistical analysis

All data analyses were performed with SPSS version 16 software (SPSS, Inc., Chicago, IL, USA). All results are expressed as mean ± standard deviation (SD). Statistical analyses were evaluated using one way analysis of variance followed by Dunnett’s test. P<0.05 was considered to indicate a statistically significant result. All experiments were performed in triplicate.

## Results

### Inhibitory effect of CDDP on HepG2 cell growth

**Figure 1:**
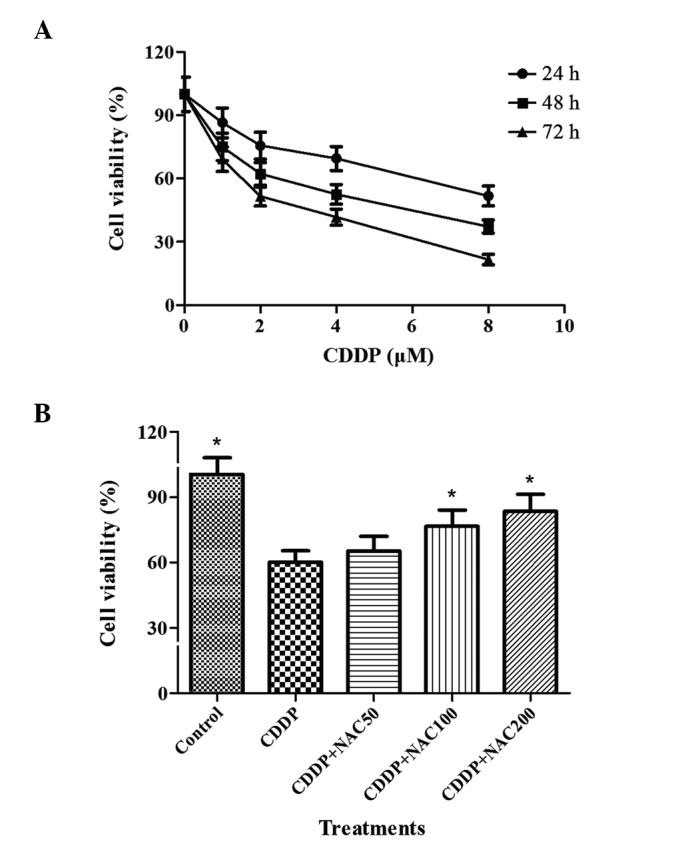
Impact of the combination of CDDP and NAC on the viability of HepG2 cells. (A) HepG2 cells were incubated with 0–8 μM CDDP for 24, 48 and 72 h, prior to the analysis of viability using the MTT assay as described in Materials and methods. For each experimental point, three cultures were prepared in parallel in different 96-well microplates with eight replicates per microplate. (B) Different concentrations of NAC were added for 3 h prior to treatment with 2 μM CDDP for 48 h. Bars indicate the means ± standard deviation of results obtained from three independent cultures per experimental point. Cell viability assessment was as described in Materials and methods. ^*^P<0.05, vs. the CDDP group, as determined by one way analysis of variance and Dunnett’s test. CDDP, cisplatin; NAC, *N*-acetylcysteine; MTT, 3-(4,5-dimethylthiazol-2-yl)-2,5-diphenyltetrazolium bromide.

HepG2 cells were incubated with various concentrations of CDDP for 24, 48 and 72 h. The viability of the cells was determine by MTT assay. The viability of the negative control was designated as 100%, and the viability of each experimental culture was expressed as a percentage compared with the negative control. The results obtained are shown in Fig. 1A. CDDP decreased the survival of the cells in a concentration-dependent manner. Following exposure to CDDP at concentrations of 1.0 μM or higher for 24 h, the cellular viability decreased compared with that of the negative control group (P<0.05). In addition, the viability was markedly reduced following 48 and 72 h of treatment with concentrations of CDDP from 1.0 to 8.0 μM compared with that of the control cells (P<0.01).

**Figure 2:**
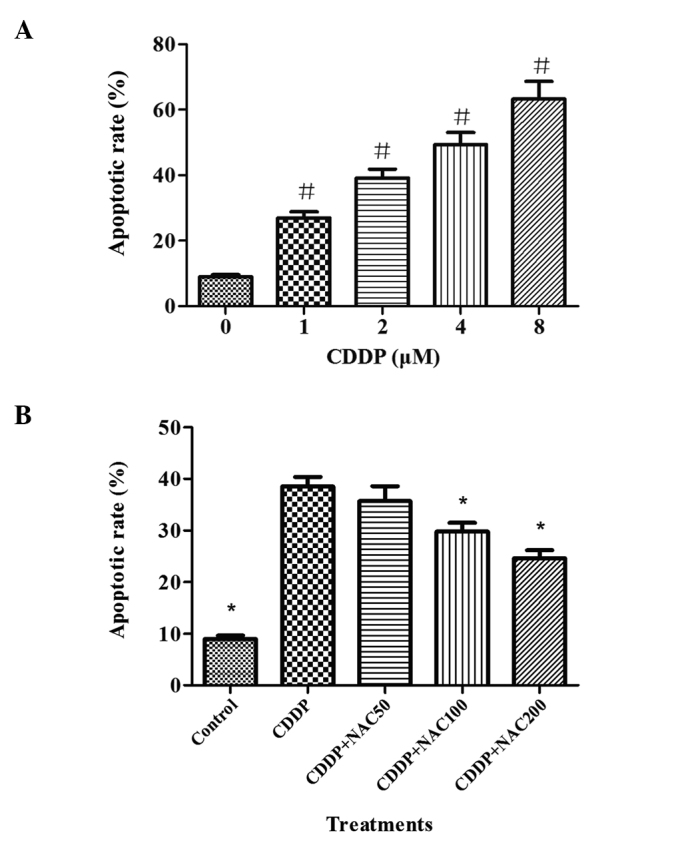
Effects of CDDP and NAC on HepG2 cell apoptosis as evaluated by flow cytometry. Cells were exposed to 0, 1, 2, 4, 8 μM of CDDP for 48 h. Then, a flow cytometric assay was carried out for the detection of apoptotic cells. NAC pretreatment was conducted at concentrations of 50, 100 and 200 μM for 3 h prior to exposure to 2 μM CDDP in HepG2 cells. (A) The apoptotic rate after exposure to various concentrations of CDDP for 48 h. (B) Apoptotic rate following NAC pre-treatment for 3 h and subsequent incubation with 2 μM CDDP for 48 h. Bars indicate the means ± SD of results obtained from three independent cultures per experimental point. The apoptosis assay was conducted as described in Materials and methods. ^#^P<0.05, vs. the control group. ^*^P<0.05, vs. the CDDP group, as determined by one way analysis of variance and Dunnett’s test. CDDP, cisplatin; NAC, *N*-acetylcysteine.

Apoptosis induced by CDDP in HepG2 cells was evaluated by flow cytometry. In the flow cytometric study, the early apoptotic and late apoptotic cells were labeled by PI in red or FITC in green. As shown in Fig. 2A, the apoptotic cell population, which was displayed on the scatter plot in red or green or both colors, increased from 26.9 to 63.3% as the concentration of CDDP increased from 1.0 to 8.0 μM for the 48-h treatment. Significant elevations were observed for the apoptotic cell population as the CDDP concentration increased.

### HepG2 cell apoptosis contributes to DNA damage

The results concerning the impact of treatment with CDDP on DNA stability in HepG2 cells are shown in Table I. The evident DNA damage induced by CDDP, as indicated by the tail length, markedly increased even at a low concentration (1.0 μM). Upon exposure to CDDP, the tail length, tail moment and olive tail moment increased in a concentration-dependent manner in the HepG2 cells compared with control group. DNA damage appeared more frequently in cells treated with higher concentrations of CDDP than in cells treated with lower ones. Marked increases were observed in tail length, tail moment and olive tail moment at higher concentrations compared with the control and lower concentrations.

### Oxidative stress damage is induced by CDDP in HepG2 cells

**Figure 3:**
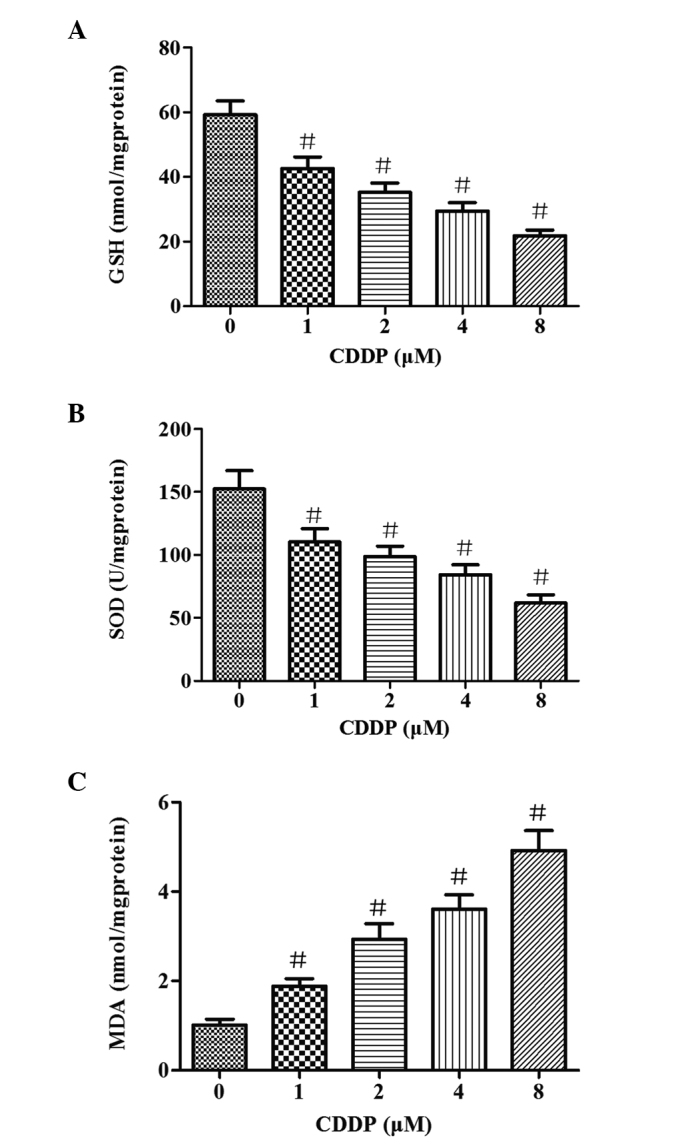
Impact on GSH, MDA and SOD levels in HepG2 cells of exposure to different concentrations of CDDP (1, 2, 4 and 8 μM) for 48 h. (A) GSH levels, (B) SOD activity and (C) MDA levels following the exposure of cultured cells to CDDP. Bars represent the means ± SD of results obtained with three independent cultures per experimental point. GSH, MDA and SOD levels were determined as described in Materials and methods. ^#^P<0.05, vs. the control group, as determined by one way analysis of variance and Dunnett’s test. GSH, glutathione; MDA, malondialdehyde; SOD, superoxide dismutase; CDDP, cisplatin.

To evaluate the effects of CDDP on oxidative stress damage in HepG2 cells, the contents of MDA and GSH were determined, as well as the activity of SOD. As shown in Fig. 3, different concentrations of CDDP led to significant (P<0.05) reductions of GSH content and SOD activity compared with that in the control group. The levels of GSH and SOD activity were inversely associated with the concentration of CDDP. When the cells were treated with various concentrations of CDDP (1–8 μM) for 48 h, the MDA content increased from 1.9 to 4.9 nmol/mg protein. These results suggest that CDDP induces oxidative damage in HepG2 cells.

### NAC protects HepG2 cells against CDDP-induced growth suppression

Following pretreatment of the HepG2 cells for 3 h with various concentrations of NAC prior to exposure to CDDP, the cell viability was determined by an MTT assay. The results are presented in Fig. 1B. They show that NAC was able to attenuate the growth suppression of HepG2 cells induced by CDDP. The survival rate of the cells that were pre-treated with NAC was higher than that of cells treated with CDDP alone. However, the viability of cells was not affected by a low concentration of NAC. Following pretreatment of the cells with 50, 100 and 200 μM of NAC, the cellular viability increased by 5.1, 16.5 and 23.2%, respectively, compared with that in the cells that were not pretreated with NAC.

The apoptotic rate of HepG2 cells, determined by staining with annexin V-FITC/PI and flow cytometric analysis is shown in Fig. 2B. The apoptotic rate was 39.2% in cells treated with 2 μM CDDP for 48 h. Following pretreatment with different concentrations of NAC (50, 100 and 200 μM), the apoptotic rates reduced to 35.7, 29.8 and 24.6%, respectively. These results indicate that NAC protected apoptosis in a concentration-dependent manner.

### NAC ameliorates CDDP-induced DNA damage

**Figure 4:**
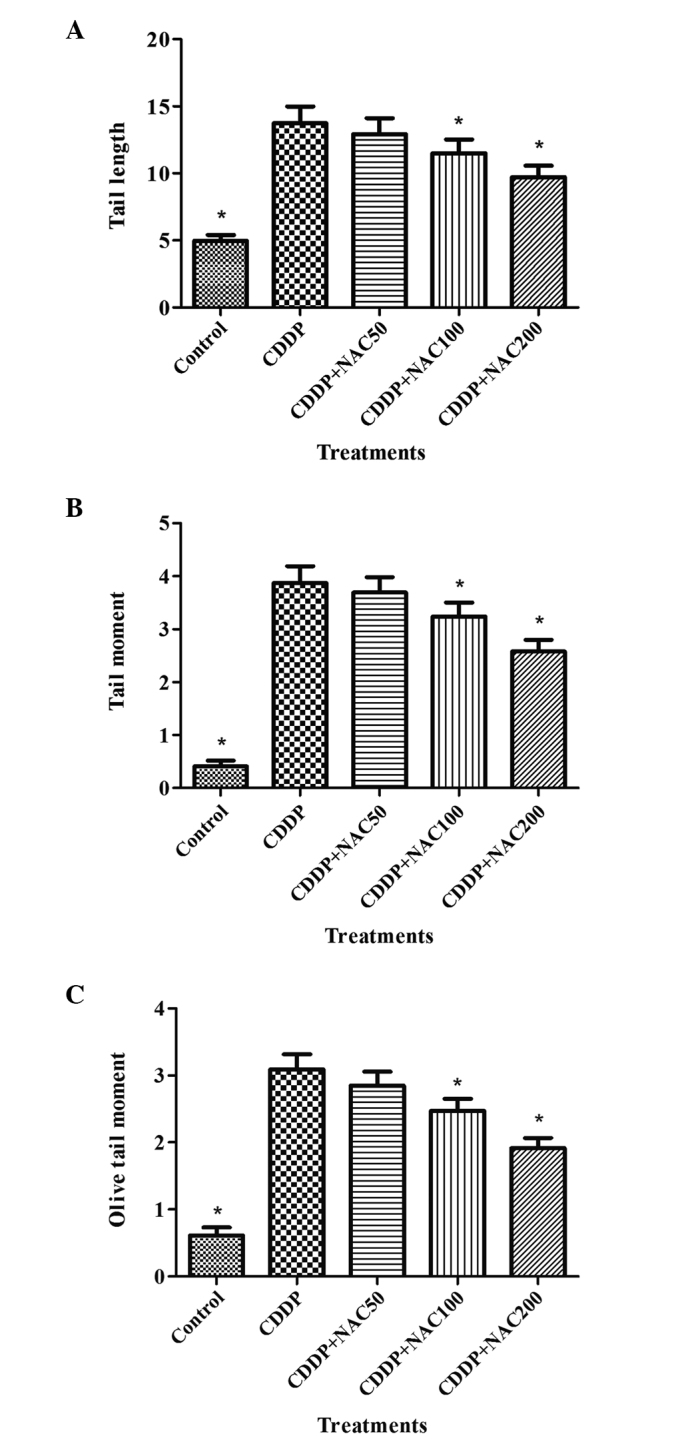
Effect on DNA damage in HepG2 cells of pretreatment with different concentrations of NAC for 3 h prior to exposure to 2 μM CDDP. (A) Tail lengths, (B) tail moments and (C) olive tail moments of cells pre-treated with different concentrations of NAC. Bars represent the means ± SD. Two slides were prepared and 100 cells were analyzed for comet formation on each. ^*^P<0.05, vs. the CDDP group, as determined by one way analysis of variance and Dunnett’s test. NAC, *N*-acetylcysteine; CDDP, cisplatin.

To further investigate the mechanism by which NAC protected the HepG2 cells from growth inhibition, DNA damage was evaluated by SCGE assay. The results of experiments in which NAC was applied to protect cells against CDDP-induced DNA damage are summarized in Fig. 4. Longer DNA tail lengths, tail moments and olive tail moments were observed in cells exposed to CDDP compared with the control group. Data for tail length, tail moment and olive tail moment in the CDDP-treated cells that were pretreated with 50 μM NAC revealed a slight reduction of migration compared with that of the cells treated with CDDP alone, although the difference was not statistically significant (P>0.05). However, treatment with 100 or 200 μM NAC prior to exposure to CDDP resulted in a statistically significant reduction of migration (P<0.05). NAC showed a protective effect on DNA damage.

### Protective effects of NAC against oxidative damage

The GSH and MDA concentrations and SOD activity in the cells are presented in Table II. The results show that CDDP (1 μM) caused significant reductions of the cellular levels of GSH and SOD compared with those in the control. Pretreatment of the cells with NAC for 3 h restored the GSH and SOD levels in a concentration-dependent manner. Exposure of the cells to CDDP resulted in a significant increase in the formation of MDA, which was attenuated when the cultures were supplemented with NAC.

## Discussion

CDDP, an alkylating agent used for the treatment of various solid tumors, is a known inducer of DNA damage. Thus, strategies for minimizing CDDP toxicity are of clinical interest. In the present study, the aim was to investigate the protective role of NAC against CDDP-induced injury in cultured HepG2 cells. The results demonstrated that CDDP inhibited cell growth, in addition to inducing cell apoptosis and DNA double-strand breaks. NAC was shown to ameliorate CDDP-induced DNA damage and oxidative stress in the HepG2 cells.

In the present study, treatment of the cells with 1 μM CDDP resulted in a significant reduction of cell viability. CDDP exerts cytotoxicity by its ability to form complexes with nucleic acids when ligand dissociation from CDDP occurs, leaving a reactive complex that interacts with deoxyribonucleic acid. Subsequently, CDDP causes inter- and intra-strand crosslinks, probably between N^7^ and O^6^ of the adjacent guanine molecules, which results in local denaturing of the DNA chain (19,20). This eventually leads to cellular death via apoptotic or non-apoptotic pathways. The DNA damage induced by CDDP is revealed by the SCGE data in the present study. A bigger DNA tail area and a longer DNA tail length compared with those of the control group were identified following the exposure of the HepG2 cells to CDDP. A recent study also reported an increase of comet formation following the treatment of HepG2 cells with platinum compounds at similar concentrations to those of CDDP used in the present study (21). Pretreatment with NAC was able to reduce the DNA injury that was observed in the CDDP group. NAC significantly decreased the cytotoxicity of CDDP due to a protective effect against DNA damage. When the cells were exposed to NAC, the apoptotic and necrotic cell populations were clearly reduced. Also, in this experiment, a clear concentration-dependent effect was observed. Apoptosis of cells is closely associated with DNA damage. Serpeloni *et al* have previously shown that lutein protects against CDDP-induced DNA damage in HepG2 cells and increases the survival rate of the cells (21).

Mistry *et al* (22) hypothesized that the detachment of the chlorines from CDDP, resulting in the platinum becoming positively charged, would attract the electronegative sulfur moiety of GSH. GSH-CDDP conjugates have been isolated from cells treated with CDDP and from the serum of CDDP-treated rats (23). In the present study, it was observed that NAC reduced CDDP-induced cell apoptosis. NAC, as a precursor of GSH, is one of the most important low molecular weight antioxidants *in vivo*. It has been suggested NAC may block the CDDP-dependent oxidation of intracellular GSH (24,25). The increased concentration of GSH is likely to have attenuated the accumulative DNA damage caused by platinum in the HepG2 cells.

Oxidative stress is involved in the toxicities of CDDP. A number of studies have demonstrated the contribution of several ROS in CDDP-induced cell damage (26,27).

For a clearer elucidation of the possible protective properties of NAC, a number of biochemical components associated with the redox status of cells were tested. SOD and GSH are important in the antioxidant defense system, while MDA is regarded as a major marker of lipid peroxidation in tissue. Lipid peroxidation is a consequence of impaired activities of antioxidant enzymes and GSH. GSH, which is a tripeptide, acts as a key ROS scavenger to protect cells in the liver against oxidative stress, and its depletion in hepatic cells could endanger the antioxidant defense system, leading to the accumulation of ROS (28). The present study shows that the GSH content of the HepG2 cells was reduced following exposure to CDDP. The results indicate that CDDP may induce lipid peroxidation products (LPO) by exhausting GSH, leading to the formation of pro-mutagenic exocyclic DNA adducts. The data in the present study regarding markers associated with oxidative stress in the CDDP-treated cells are concordant with the findings of a previous study (29). NAC exhibits antioxidative properties through increasing the concentrations of GSH in cells.

SOD, a scavenger of superoxide, is the most important protective enzyme that provides the first line of enzymatic antioxidant defense against oxidative stress in various organs and tissues (30–32), including the liver. It provides protective effects against oxygen free radicals as it catalyzes the removal of the superoxide radical (O^2•−^), which damages membranes and biological structures. In the present study, it was found that CDDP significantly decreased the SOD level in HepG2 cells in a concentration-dependent manner. Exposure to CDDP at 1–8 μM for 48 h resulted in a significant reduction of SOD activity, whereas NAC pre-treatment partially reversed this effect.

SOD activity and MDA concentration are usually measured together. MDA is one of the end-products of membrane lipid peroxidation (33). It has been postulated that the mechanism of peroxidation involves the formation of prostaglandin-like endoperoxides from polyunsaturated fatty acids that have two or more double bonds (34). Therefore, in the present study, the MDA concentration was determined as another marker of oxidative damage. The MDA level significantly increased compared with that of control group when the cells were treated with various concentrations of CDDP. This result indicates that lipid peroxidation products were produced as well as DNA damage in the cells. Pretreatment of the cells with NAC for 3 h prior to exposure to 2 μM CDDP, resulted in the MDA levels being reduced.

A reduction or loss of antioxidant enzyme activity in HepG2 cells may result in oxidative stress and induce lipid peroxidation, DNA damage, and cell injury, and subsequent cell death. Leonardo *et al* (35) demonstrated that PC12 cells treated with CDDP exhibited an increased generation of ROS, which may be one of the main mechanisms by which CDDP exerts DNA genotoxicity. In CDDP-induced renal damage, the increased production of ROS can lead to an enhancement in the expression of proinflammatory mediators, which could intensify the cytotoxic effects (36). In a previous study it was found that the exposure of cells to 2,4,5,2′,4′,5′-hexachlorobiphenyl (PCB153) decreased SOD activity and the concentration of MDA and increased cell apoptosis, and that NAC pretreatment had a protective effect as it significantly reduced the cell apoptosis (37). In the present study, increased MDA levels and reduced GSH levels and SOD activity were observed in association with CDDP-induced cytotoxicity. The imbalance of the intracellular antioxidant/oxidant system exacerbated HepG2 cell apoptosis. NAC treatment was able to ameliorate the oxidative stress and cytotoxicity.

In summary, the results of the present study suggest that CDDP induces pronounced oxidative stress in HepG2 cells that is associated with DNA damage, and that supplementation with the antioxidant NAC may protect against these adverse effects caused by the platinum compound. These findings suggest that NAC may be a useful supplement in chemotherapy involving platinum compounds.

## Figures and Tables

**Table I:** Results of the single cell gel electrophoresis assay after exposure of cultured HepG2 cells to cisplatin for 48 h.

Concentration (μM)	Tail length	Tail moment	Olive tail moment
0	5.16±1.03	0.43±0.13	0.57±0.19
1	13.97±2.11^a^	2.55±0.27^a^	2.03±0.36^a^
2	19.81±2.73^a^	3.82±0.39^a^	3.15±0.42^a^
4	34.94±4.28^a^	6.29±0.52^a^	5.19±0.53^a^
8	60.15±6.46^a^	11.73±1.04^a^	9.61±0.89^a^

Data are expressed as the mean ± SD. One hundred nucleoids were analyzed per treatment.

a P<0.01 vs. the control (0 μM) group.

**Table II:** Results of pre-treatment with NAC for 3 h prior to incubation with CDDP (2 μM) in HepG2 cells.

NAC concentration (μM)	GSH (nmol/mg protein)	SOD (U/mg protein)	MDA (nmol/mg protein)
0	34.82±2.74	97.92±8.58	2.87±0.33
50	39.14±2.91^a^	112.46±9.73^a^	2.42±0.31^a^
100	42.27±3.17^b^	119.32±9.52^b^	2.03±0.27^b^
200	46.38±3.29^b^	126.75±10.04^b^	1.79±0.19^b^

NAC, *N*-acetylcysteine; CDDP, cisplatin; GSH, glutathione; MDA, malondialdehyde; SOD, superoxide dismutase. Data are expressed as the mean ± SD.

a P<0.05,

b P<0.01 vs. the negative control.
